# TOP2A Overexpression as a Prognostic Biomarker in High-Grade Meningioma: An Integrated Single-Cell and Bulk Transcriptomic Study

**DOI:** 10.3390/ijms27208984

**Published:** 2026-10-10

**Authors:** Hongqing Cai, Guanchao Xie, Houjie Liu, Yujia Chen, Huaixu Li, Jinghai Wan, Zekun Deng

**Affiliations:** Department of Neurosurgery, National Cancer Center/National Clinical Research Center for Cancer/Cancer Hospital, Chinese Academy of Medical Sciences and Peking Union Medical College, No. 17 Panjiayuan Nanli, Chaoyang District, Beijing 100021, China; phonecy@126.com (H.C.);

**Keywords:** meningioma, TOP2A copy-number gain, prognostic biomarker

## Abstract

While prior single-cell studies have identified proliferative neoplastic subpopulations, the prognostic and therapeutic implications of TOP2A focal copy-number status have not been defined. This study aimed to delineate the cellular architecture of high-grade meningioma and to evaluate the clinical and prognostic significance of TOP2A expression together with its focal copy-number status through integrated single-cell and bulk transcriptomic profiling. Single-cell RNA sequencing (scRNA-seq) was integrated with bulk RNA sequencing (bulk RNA-seq) to resolve the cellular landscape of high-grade meningioma in an exploratory cohort, followed by functional subcluster identification, differential expression analysis, and survival analyses. TOP2A was prioritized for multi-level validation: mRNA overexpression was confirmed in an independent bulk cohort, protein overexpression by immunohistochemistry (IHC), and focal gene copy-number status by fluorescence in situ hybridization (FISH). Kaplan–Meier survival analysis was performed to evaluate patient prognosis. Correlations between continuous variables were assessed using Spearman’s rank correlation coefficient. scRNA-seq of an exploratory high-grade cohort revealed a cell-cycle proliferation cluster characterized by high TOP2A expression. In a 160-sample bulk RNA-seq cohort, TOP2A was overexpressed in high-grade and recurrent tumors, and elevated TOP2A mRNA was significantly associated with shorter progression-free survival (PFS). IHC on 154 specimens confirmed TOP2A protein overexpression, and FISH on 27 specimens demonstrated focal TOP2A copy-number gain enriched in high-grade cases. Copy-number gain was concordant with TOP2A mRNA levels. Although TOP2A mRNA and protein overexpression were each associated with shorter PFS, the limited size of the FISH subset precluded an independent survival analysis by copy-number status. This study identifies TOP2A mRNA and protein overexpression as a prognostic biomarker in high-grade meningioma, and shows that focal TOP2A copy-number gain is enriched in high-grade tumors and concordant with TOP2A expression. By integrating transcriptional overexpression with copy-number gain, our findings identify TOP2A as a candidate prognostic biomarker warranting prospective evaluation in high-grade meningioma.

## 1. Introduction

Meningioma is the most prevalent primary intracranial neoplasm, comprising approximately 37% of all primary brain tumors [1]. Its global age-standardized annual incidence varies from 1.3 to 7.8 per 100,000 population [2]. According to the 2021 World Health Organization (WHO) classification, CNS WHO Grade 1 tumors account for 80.1% of cases, Grade 2 for 18.3%, and Grade 3 for 1.5% [3].

Current therapeutic strategies for meningioma face distinct challenges across the disease spectrum. While complete surgical resection achieves cure rates exceeding 90% for benign WHO Grade 1 tumors [4], and radiotherapy or stereotactic radiosurgery provides 80–90% 10-year local control for unresectable or residual lesions [4], managing high-grade or recurrent meningiomas remains formidable due to their aggressive biology and high recurrence rates. The development of systemic pharmacotherapies is further hindered by limited understanding of tumor cellular architecture and the immune microenvironment, coupled with a paucity of positive randomized controlled trials [5]. Notably, despite maximal safe resection combined with adjuvant radiotherapy, patients with WHO Grade 2 and Grade 3 meningiomas experience significantly poorer outcomes, with 5-year progression-free survival rates plummeting to 48% and 13%, respectively [4], while recurrent high-grade tumors often exhibit treatment resistance, leaving patients with limited therapeutic options and a median overall survival of less than 2 years following multiple recurrences [6]. Consequently, elucidating the molecular landscape of meningioma has become a critical imperative to address these unmet therapeutic needs. Recent technological advancements, particularly in single-cell sequencing, have invigorated tumor microenvironment (TME) research. By generating high-resolution profiles of tumor specimens, this technology facilitates the systematic characterization of intratumoral heterogeneity and elucidates the compositional architecture and molecular landscape of cellular constituents.

Recent single-cell and spatial transcriptomic studies have advanced our understanding of meningioma cellular heterogeneity [7,8,9]. Notably, a small MKI67/TOP2A-positive proliferative neoplastic subpopulation has been identified in atypical and transitional meningiomas [8], and a large-scale multi-omic study has defined cell states associated with progression and outcome [10]. However, the prognostic significance of TOP2A focal copy-number status and its therapeutic implications have not been defined. In this study, we applied integrated single-cell and bulk transcriptomic approaches to high-grade meningioma specimens and performed multi-level validation of TOP2A-including mRNA overexpression, protein overexpression, and focal gene copy-number gain-to evaluate its potential as a candidate prognostic and therapeutic biomarker. We identified distinct cell subtypes and molecular signatures linked to malignant progression, and we provide FISH-based evidence that focal TOP2A copy-number gain is enriched in high-grade tumors and concordant with TOP2A mRNA expression. Collectively, these results establish a framework for TOP2A-guided risk stratification and the discovery of meningioma-directed therapeutic targets.

## 2. Results

### 2.1. Single-Cell Characterization of High-Grade Meningioma Cellular and Functional Architecture in an Exploratory Cohort

**Figure 1:**
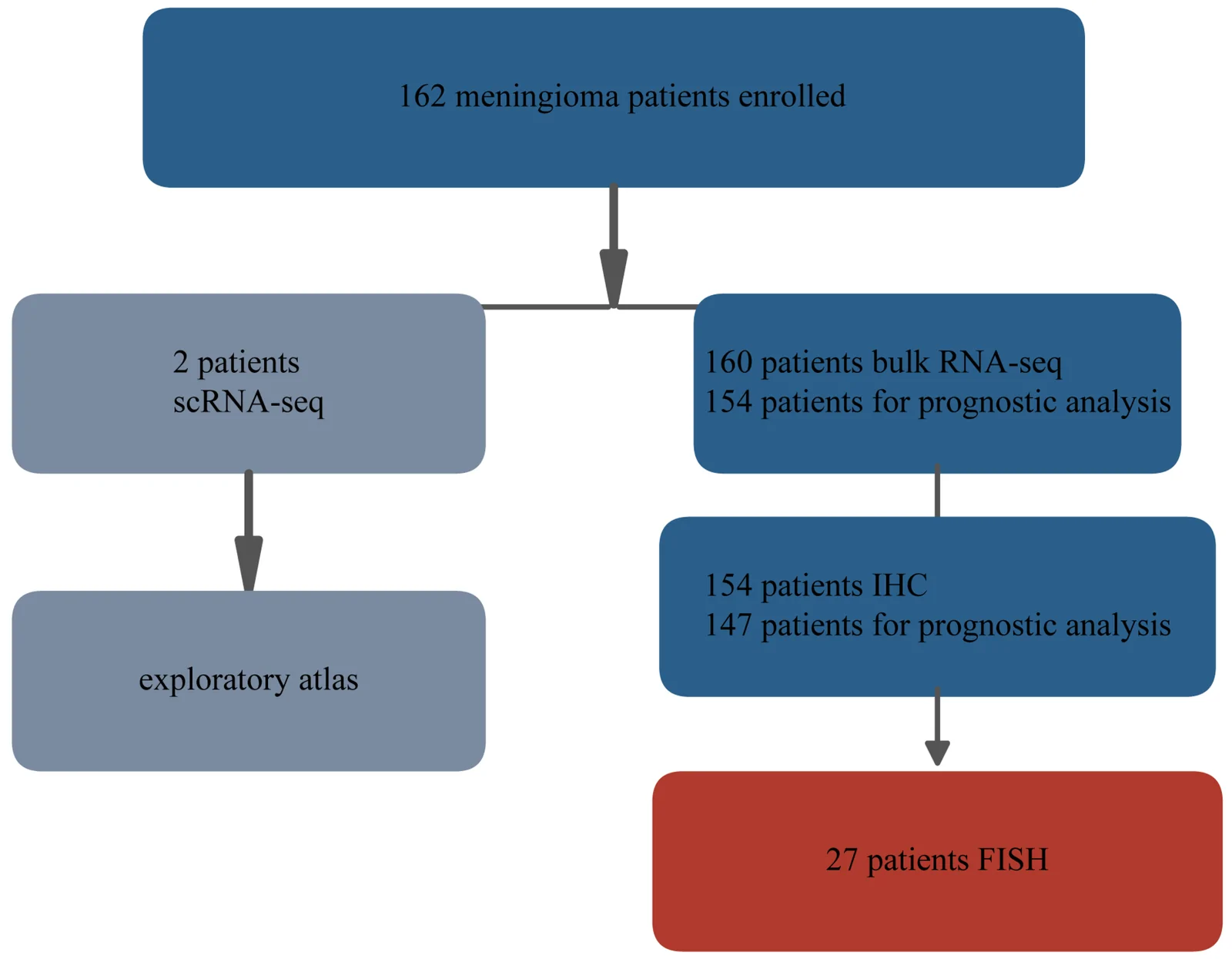
Study workflow and specimen flow diagram, showing the 162 enrolled patients and the 160/154/147 subsets analysed at each step. Coloured boxes denote the successive analysis modules and arrows indicate the flow of specimens and data.

**Figure 2:**
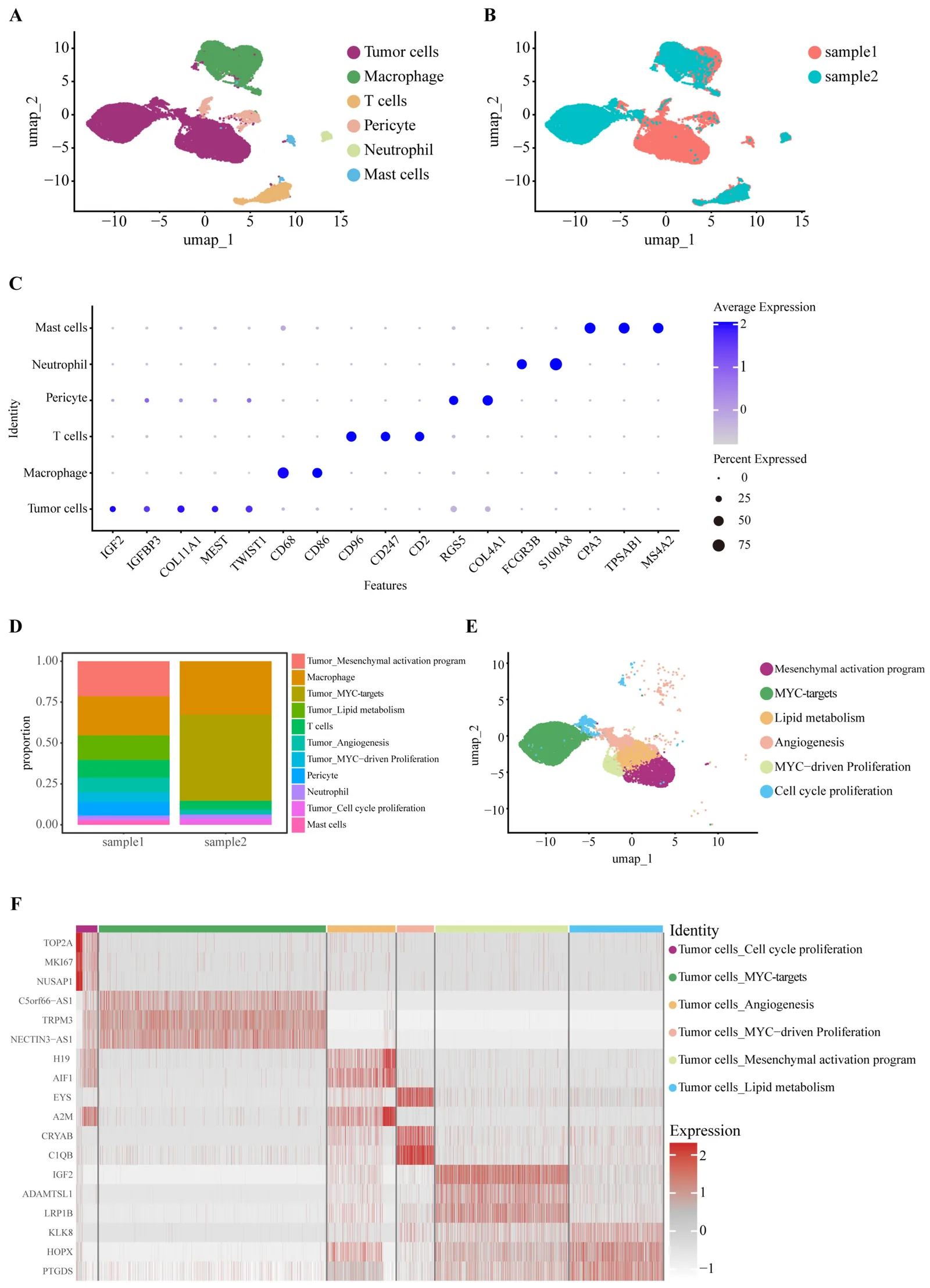
Single-cell transcriptomic landscape reveals the cellular architecture and functional heterogeneity of meningioma. (**A**) Global UMAP visualization of 25,635 single cells, color-coded by six major cell lineages: Tumor cells, Macrophages, T cells, Pericytes, Neutrophils, Mast cells. (**B**) UMAP plot displaying the distribution of cells from the two patient samples, plotted by sample of origin. Cells from both specimens occupy the same lineage-defined regions; with only two patients, however, this pattern cannot distinguish successful batch correction from genuine inter-patient heterogeneity. (**C**) Dot plot illustrating the expression of canonical lineage-specific marker genes used for cell type annotation. Dot size represents the percentage of cells expressing each gene, and color intensity indicates average expression level. (**D**) Stacked bar plots showing the relative composition of cell states in sample1 and sample2. Each bar corresponds to one sample and is normalized to 100%; the height of each segment indicates the percentage of that population within the total cells of that sample. Non-malignant cells are annotated by their canonical lineage identity (macrophages, T cells, pericytes, neutrophils and mast cells). Tumor cells, in contrast, are not treated as a single homogeneous population but are instead partitioned into distinct transcriptional/functional programs according to their dominant expression state—cell cycle proliferation, MYC-driven proliferation, angiogenesis, mesenchymal activation, MYC targets and lipid metabolism. The malignant compartment is subdivided by function rather than by lineage, whereas all non-malignant compartments remain lineage-based definitions. (**E**) UMAP visualization of the tumor compartment, stratified into six functional subclusters based on Gene Set Variation Analysis (GSVA): Cell cycle proliferation, MYC-driven proliferation, Angiogenesis, Mesenchymal activation program, MYC-targets, and Lipid metabolism. (**F**) Heatmap displaying the top three differentially expressed genes (ranked by log_2_ Fold Change) for each functional tumor subcluster. Side bars indicate functional subcluster identity (top) and scaled gene expression (right).

The workflow plot of this study is presented in Figure 1. The clinical profiles of the two patients whose tumor specimens were subjected to scRNA-seq profiling are summarized in Supplementary Table S1. This two-case scRNA-seq cohort was designed as an exploratory, hypothesis-generating atlas of high-grade meningioma rather than a representative survey across the full disease spectrum. The corresponding preoperative T1-weighted contrast-enhanced magnetic resonance imaging (MRI) scan of these two cases are presented in Supplementary Figure S1. Single-cell RNA sequencing was performed on two meningioma specimens, and the resulting cellular landscape comprising 25,635 single cells was visualized using Uniform Manifold Approximation and Projection (UMAP). All cells resolved into six major cell lineages: Tumor cells, Macrophages, T cells, Pericytes, Neutrophils and Mast cells (Figure 2A). In sample 1 (15,115 cells), tumor cells were most abundant (7967; 52.71%), followed by macrophages (3595; 23.78%), T cells (1623; 10.74%), pericytes (1245; 8.24%), neutrophils (344; 2.28%), and mast cells (341; 2.26%). Sample 2 (10,520 cells) was likewise dominated by tumor cells (6212; 59.05%) and macrophages (3436; 32.66%), followed by T cells (548; 5.21%), neutrophils (277; 2.63%), pericytes (25; 0.24%), and mast cells (22; 0.21%). Cells from both specimens occupied the same lineage-defined regions after annotation by sample of origin (Figure 2B), subject to the limitation that with only two patients a residual batch effect cannot be fully distinguished from genuine inter-patient heterogeneity. Canonical lineage markers were expressed in a lineage-restricted manner, confirming the annotation (Figure 2C; Supplementary Material S1). CNV analysis confirmed the malignant identity of the tumor compartment (Supplementary Figure S2).

Whereas the lineage composition above describes the tumor as a static cellular census, its malignant potential ultimately resides in functional state. To dissect this functional diversity, Gene Set Variation Analysis (GSVA) was applied to the tumor cells (Supplementary Figure S3), stratifying them, based on distinct pathway-enrichment profiles, into six functionally discrete subpopulations; the seven primary clusters resolved by this analysis were reduced to six by combining the two that lay closest to each other in the UMAP embedding (g3 and g4). The proportional distribution of all subpopulations, including malignant and non-malignant cells, is quantified in Figure 2D, revealing pronounced sample-specific compositional differences. The UMAP distribution of these subpopulations delineated six functional subsets—Cell cycle proliferation, MYC-driven proliferation, Angiogenesis, Mesenchymal activation program, MYC-targets, and Lipid metabolism (Figure 2E). This functional stratification revealed that high-grade meningioma cells are heterogeneous rather than uniform—proliferative programs coexisting with mesenchymal, angiogenic, and metabolic programs within the same tumor—a diversity with direct therapeutic implications, as functionally distinct subpopulations may respond differently to any single agent. The complete ranked marker lists for all six functional subpopulations are provided in Supplementary Material S2, and the top three markers of each subpopulation are displayed as a heatmap (Figure 2F). Among these programs, the top-ranked marker of the Cell cycle proliferation subcluster was TOP2A (ranked by log_2_ Fold Change (logFC); Figure 2F), a well-established proliferation marker previously reported in MKI67/TOP2A-positive proliferative subpopulations of meningioma [8]. As focal copy-number amplification is a common mechanism by which such proliferative drivers become activated and sustained, TOP2A was carried forward for downstream multi-level prognostic validation. Because this two-case scRNA-seq cohort was designed as an exploratory, hypothesis-generating atlas rather than a representative survey of the full disease spectrum, these observations were subsequently tested in an independent bulk transcriptomic cohort. Because the cell-cycle subpopulation was defined by cell-cycle pathway enrichment, high expression of cell-cycle genes within it is expected by construction; this observation was therefore not used as a criterion for prioritising TOP2A, whose selection rests on the independent bulk-cohort evidence presented below.

### 2.2. Integrated Bulk RNA-Seq Analysis and Prognostic Significance of TOP2A

**Figure 3:**
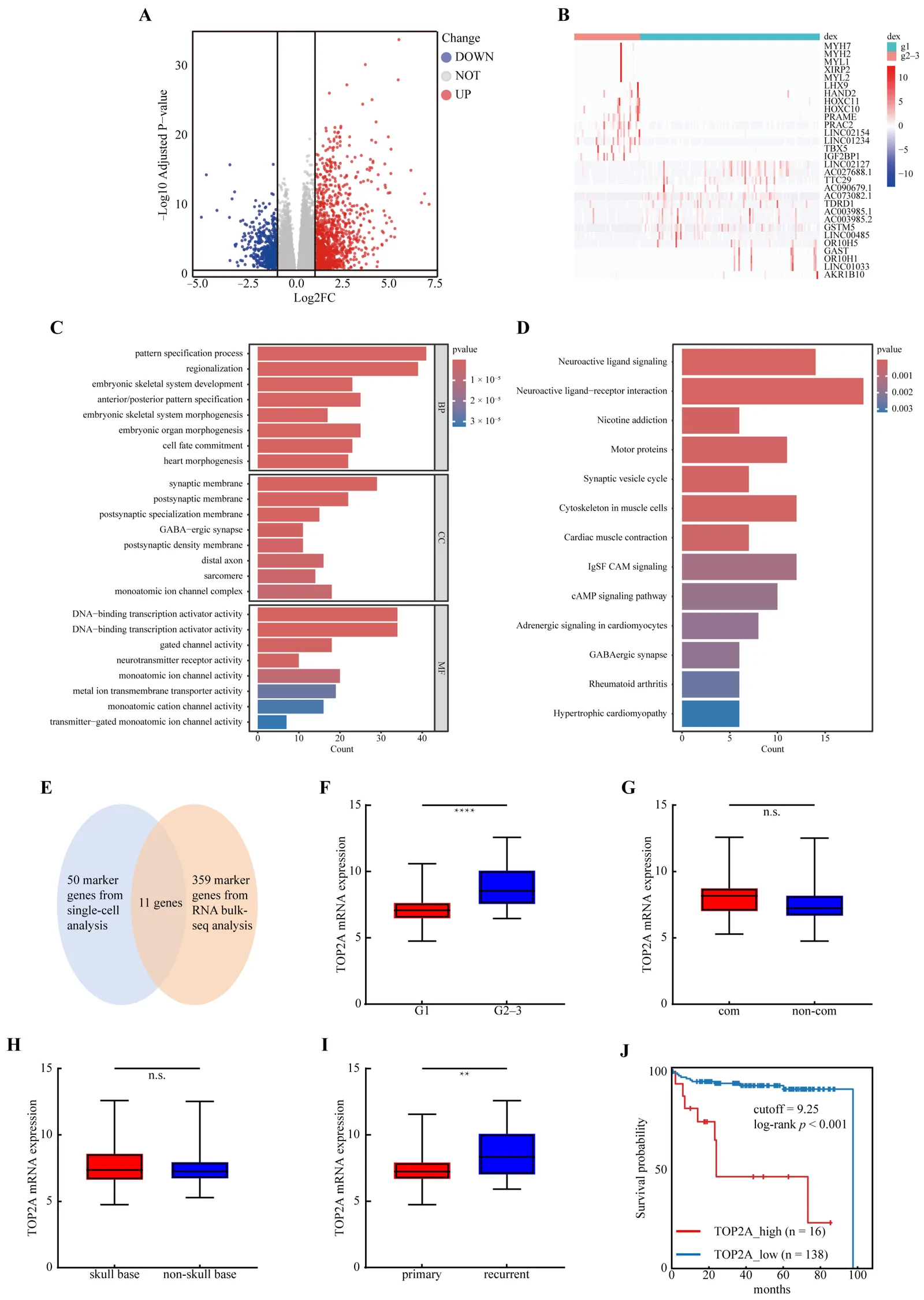
Integrated bulk RNA-seq and survival analysis reveal TOP2A as a potential oncogenic driver in meningioma. (**A**) Volcano plot of differentially expressed genes (DEGs) identified from bulk RNA-seq data of 160 meningioma patients. Red dots represent significantly upregulated genes, blue dots represent significantly downregulated genes, and gray dots represent genes with no significant change. A total of 359 DEGs were identified (300 upregulated, 59 downregulated), applying thresholds of adjusted *p* < 0.001 and |log_2_FC| > 2. (**B**) Heatmap showing hierarchical clustering of the top-ranked DEGs across patient samples stratified by tumor grade (Grade 1 vs. Grade 2–3). Red and blue indicate high and low expression, respectively. (**C**,**D**) Functional enrichment analysis of the 359 DEGs. (**C**) Bar plot of the top enriched GO biological process terms. (**D**) Bar plot of the top enriched KEGG pathways. The color scale represents statistical significance (−log_10_ *p*-value). (**E**) Venn diagram illustrating the overlap between 50 single-cell-specific marker genes and the 359 bulk RNA-seq DEGs. Eleven intersecting genes were identified; all 11 were evaluated by univariate Cox regression for progression-free survival (Supplementary Table S2), on the basis of which TOP2A was advanced for further investigation. (**F**–**I**) Box plots comparing TOP2A expression levels across clinical subgroups: (**F**) WHO tumor grade (Grade 1 vs. Grade 2–3), (**G**) extracranial extension (communicating vs. non-communicating), (**H**) skull base involvement (skull base vs. non-skull base), and (**I**) tumor recurrence status (primary vs. recurrent). *p* values were calculated using two-sided Mann–Whitney U tests and adjusted for multiple comparisons using the Benjamini–Hochberg procedure within the bulk RNA-seq family (m = 4); q values are shown in each panel. (**J**) Kaplan–Meier plot of progression-free survival (PFS) in 154 patients stratified by TOP2A mRNA expression, dichotomized at the cutoff that maximized the log-rank statistic (maximally selected rank statistic; high, log_2_ > 9.2503, *n* = 16 vs. low, log_2_ ≤ 9.2503, *n* = 138). The uncorrected log-rank *p* was 4.7 × 10^−8^, and the Lausen–Schumacher cutoff-corrected *p* was 3.0 × 10^−6^. (** q < 0.01, **** q < 0.0001; n.s., not significant.).

To validate our single-cell findings at the bulk transcriptomic level, we performed bulk RNA sequencing (RNA-seq) on 160 clinically annotated meningioma specimens, including 117 CNS WHO Grade 1, 38 Grade 2, and 5 Grade 3 cases. Differential expression analysis (Figure 3A) identified 359 differentially expressed genes (DEGs) (300 upregulated and 59 downregulated) between Grade 1 and Grade 2–3 tumors (Supplementary Material S3). Hierarchical clustering of these DEGs, with samples stratified by tumor grade, revealed distinct expression signatures, with a specific subset of genes showing elevated expression in higher-grade (Grade 2–3) tumors (Figure 3B). GO and KEGG enrichment analyses of the DEGs highlighted significant perturbations in neuroactive ligand–receptor interactions, muscle system processes, and DNA replication pathways (Figure 3C,D), suggesting enhanced proliferative capacity and altered neuroactive signaling in higher-grade tumors.

To bridge the single-cell and bulk transcriptomic data, we intersected the 359 bulk DEGs with the 50 top-ranked marker genes of the tumor-cell compartment, obtained by ranking all subpopulation markers by average log_2_ fold change (avg_log_2_FC) (Supplementary Material S2). This intersection yielded 11 overlapping genes (Figure 3E). A total of 11 candidate genes were identified, namely BIRC5, C5orf66-AS1, CHP2, FMO3, LINC01524, NLGN4X, NNAT, RBFOX1, SHISA9, SLC6A1, and TOP2A. Univariate Cox regression analysis of progression-free survival (PFS) was then performed for these 11 candidate genes in 154 meningioma patients (Supplementary Table S2). No single metric identified TOP2A unambiguously, and it was therefore prioritized on the basis of two complementary criteria. First, although its hazard ratio (HR = 1.763) ranked second among the 11 genes, the gene with the largest HR, LINC01524 (HR = 1.779), was near-undetectable in the bulk RNA-seq data (81.8% of samples with zero counts; Supplementary Material S4), as were CHP2 (61.0%) and C5orf66-AS1 (78.6%), which ranked first and second on statistical significance (Benjamini–Hochberg-adjusted q = 3.01 × 10^−6^ and 1.17 × 10^−5^, respectively, versus 2.04 × 10^−5^ for TOP2A); among the candidates detected in all 154 samples (TOP2A, BIRC5 and SLC6A1), TOP2A and BIRC5 showed statistically indistinguishable associations (per-log_2_-unit HR = 1.763, 95% CI 1.38–2.25 versus 1.738, 1.35–2.23; standardised per-SD HR = 2.27 versus 2.25; Supplementary Table S2). Because the two effect sizes do not differ meaningfully, we do not claim that TOP2A outperforms BIRC5 in our cohort. Second, TOP2A was selected over BIRC5 on the basis of its established druggability, as the direct target of approved topoisomerase II inhibitors, and the availability of validated IHC and FISH assays for TOP2A, neither of which is available for BIRC5. Accordingly, TOP2A was prioritized for subsequent analyses, including direct assessment of its copy-number status by fluorescence in situ hybridization (FISH). Analysis of TOP2A expression across clinical subgroups revealed significantly higher expression in high-grade tumors (Grade 1, *n* = 117 vs. Grade 2–3, *n* = 43; *p* = 2.09 × 10^−8^, Benjamini–Hochberg-adjusted q = 8.36 × 10^−8^; Figure 3F) and in recurrent tumors (primary, *n* = 130 vs. recurrent, *n* = 30; *p* = 3.91 × 10^−3^, Benjamini–Hochberg-adjusted q = 7.82 × 10^−3^; Figure 3I). In contrast, no statistically significant difference in TOP2A expression was observed between skull base and non-skull base tumors (skull base, *n* = 85 vs. non-skull base, *n* = 75; *p* = 0.847, Benjamini–Hochberg-adjusted q = 0.847; Figure 3H). Clinically communicating tumors showed a trend toward higher TOP2A expression (communicating, *n* = 26 vs. non-communicating, *n* = 134; *p* = 0.0440) that did not retain significance after Benjamini–Hochberg adjustment (q = 0.0586; Figure 3G); Holm step-down and Bonferroni corrections yielded concordant results. Furthermore, among the 154 patients with available follow-up data, high TOP2A mRNA expression (log_2_ > 9.2503; Supplementary Material S6) was associated with significantly shorter progression-free survival (median 24.0 months vs. not reached; HR = 8.79, 95% CI 3.45–22.41; log-rank *p* = 4.7 × 10^−8^, Lausen–Schumacher-corrected *p* = 3.0 × 10^−6^), supporting TOP2A as a potential prognostic biomarker in meningioma (Figure 3J).

To identify prognostic factors for meningioma, univariate and multivariate Cox proportional hazards regression analyses were performed using progression-free survival (PFS) as the endpoint (Supplementary Material S5). Among the 154 patients in this cohort, 19 progression events (12.3%) were observed during follow-up. In univariate analysis, higher TOP2A mRNA expression was significantly associated with shorter PFS (hazard ratio [HR] = 1.76 per log_2_ unit, 95% confidence interval [CI] 1.38–2.25, *p* = 5.6 × 10^−6^; Table 1). In the final multivariate model, TOP2A mRNA remained an independent predictor of PFS after adjustment for presentation status and Simpson grade (HR = 1.54, 95% CI 1.20–1.98, *p* = 6.4 × 10^−4^; Table 2). The model showed good discrimination (C-index = 0.887) and overall significance (likelihood ratio test, *p* = 2.6 × 10^−10^; Supplementary Table S3), with no problematic multicollinearity among the covariates (all variance inflation factors < 2.3; Supplementary Table S4). The effect of TOP2A was stable across sequential adjustment for WHO grade, presentation status, and Simpson grade (HR from 1.76 to 1.47; Supplementary Table S5), and we therefore determined whether TOP2A adds prognostic information beyond the routine proliferation marker Ki-67, entering both markers simultaneously in a model that also included presentation status and Simpson grade (Supplementary Table S10). TOP2A retained an independent association with shorter PFS (HR = 1.42, 95% CI 1.08–1.86, *p* = 1.1 × 10^−2^), whereas Ki-67 did not (HR = 1.41 per 10%, 95% CI 0.82–2.46, *p* = 0.218). Adding TOP2A to a model already containing Ki-67 significantly improved model fit (likelihood-ratio test, χ^2^ = 7.10, df = 1, *p* = 7.7 × 10^−3^; ΔAIC = −5.10; continuous NRI = +0.79) without further improving discrimination (C-index 0.897 with TOP2A vs. 0.899 without; ΔC-index = −0.002; out-of-bag bootstrap 95% CI −0.106 to +0.065), consistent with the two markers capturing largely overlapping prognostic information. The substitution analysis (Supplementary Table S6) is retained as a complementary sensitivity analysis. The proportional hazards assumption was satisfied for all covariates (Supplementary Table S7). To further assess the robustness of the association, the multivariable model was rebuilt using alternative variable-selection strategies: forward selection (entry *p* < 0.05), with or without forcing TOP2A mRNA, and backward elimination (removal *p* ≥ 0.05) all converged on the identical model comprising presentation status, Simpson grade, and TOP2A mRNA, yielding an unchanged hazard ratio for TOP2A (HR = 1.54, 95% CI 1.20–1.98, *p* = 6.4 × 10^−4^; Supplementary Table S8). We additionally fitted a sensitivity model in which TOP2A mRNA, WHO grade, Simpson grade, age, adjuvant radiotherapy and Ki-67 were all forced in irrespective of their univariate *p* value (Supplementary Table S9); TOP2A remained independently associated with shorter PFS (HR = 1.65, 95% CI 1.19–2.30, *p* = 2.6 × 10^−3^), and its estimate was stable when each other covariate was omitted in turn (HR 1.52–1.69). Because only 19 progression events were available (3.2 per variable), this model is at the lower limit of what the data can support, and bootstrap resampling gave a wider interval for the TOP2A effect (median HR = 1.67, 95% percentile CI 0.82–2.78). Collectively, these results indicate that TOP2A overexpression is an independent adverse prognostic factor for PFS in meningioma.

### 2.3. Concordance of TOP2A mRNA, Protein and Focal Copy-Number Status

**Figure 4:**
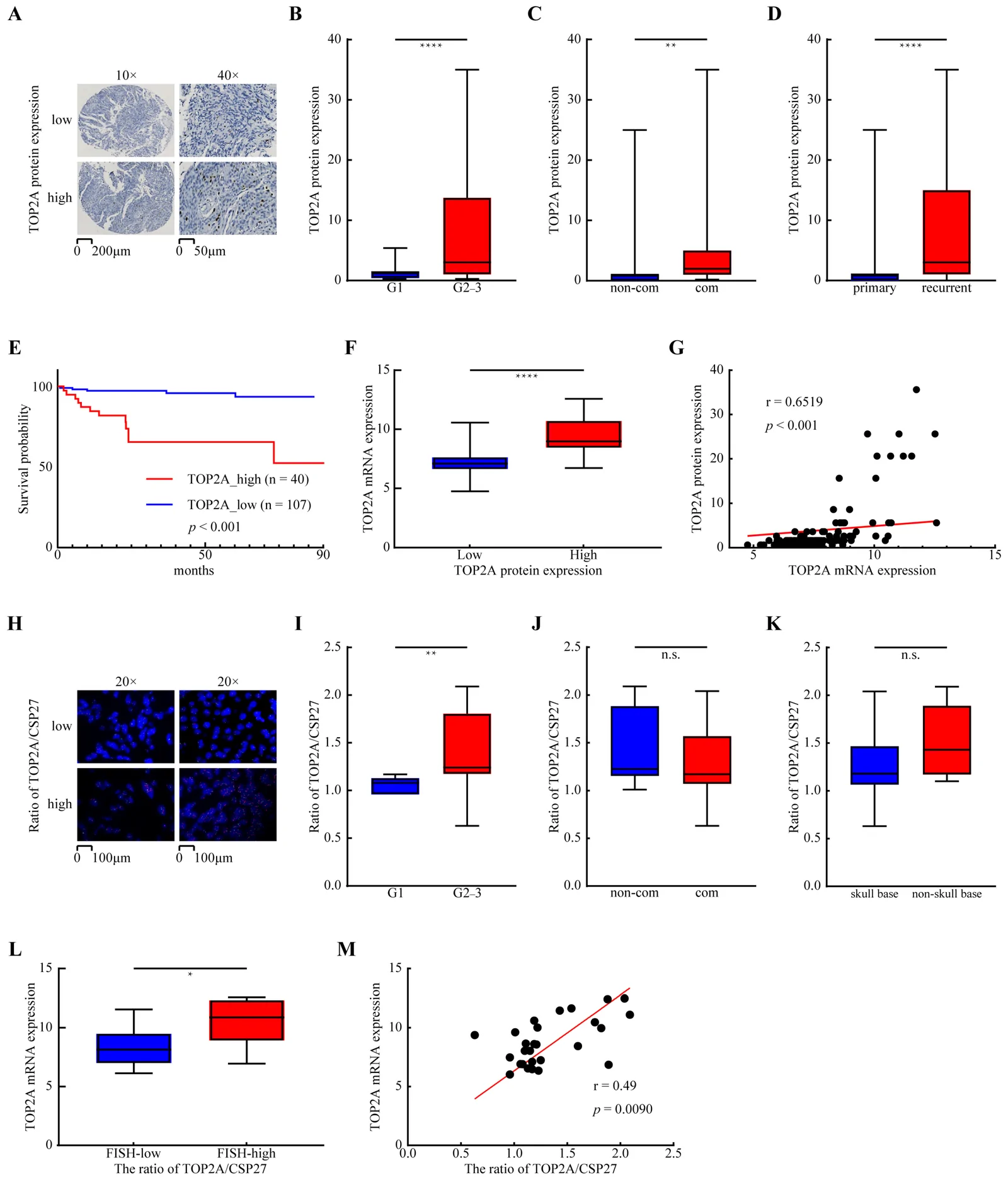
Correlation of TOP2A protein expression and focal copy-number status with mRNA levels. (**A**) Representative TOP2A immunohistochemical staining of meningioma specimens, showing markedly stronger nuclear TOP2A staining in high-grade (Grade 2–3) tumors compared to low-grade (Grade 1) tumors (scale bars: 200 μm, 10×; 50 μm, 40×). (**B**–**D**) Quantitative analysis of TOP2A protein expression (IHC H-score) in: (**B**) Grade 1 vs. Grade 2–3 tumors, (**C**) non-communicating vs. communicating subtypes, and (**D**) primary vs. recurrent cases. (**E**) Kaplan–Meier curve of progression-free survival (PFS) for 147 patients stratified by TOP2A protein expression (H-score > 1, *n* = 40 vs. H-score ≤ 1, *n* = 107), dichotomized at the cutoff that maximized the log-rank statistic (maximally selected rank statistic). The uncorrected log-rank *p* was 1.75 × 10^−6^, and the Lausen–Schumacher cutoff-corrected *p* was 9.1 × 10^−5^. (**F**) TOP2A mRNA expression (FPKM) in the protein-high vs. protein-low groups (Mann–Whitney U test, *p* = 1.6 × 10^−12^). (**G**) Scatter plot of TOP2A mRNA (FPKM) versus protein (IHC H-score) expression across the matched specimens, with Spearman correlation (r = 0.65, 95% CI 0.54–0.74, *p* = 3.8 × 10^−19^). (**H**) Representative dual-color FISH images of TOP2A (red) and chromosome 17 centromere (CSP27, green) in meningioma samples, showing elevated TOP2A/CSP27 ratios in cases with high-level amplification (scale bars: 100 μm). (**I**–**K**) Quantitative analysis of the TOP2A/CSP27 ratio in: (**I**) Grade 1 vs. Grade 2–3 tumors, (**J**) non-communicating vs. communicating subtypes, and (**K**) skull base vs. non-skull base cases. (**L**) TOP2A mRNA expression (FPKM) in the FISH-high (TOP2A/CSP27 ratio ≥ 1.5, *n* = 8) vs. FISH-low (ratio < 1.5, *n* = 19) groups (Mann–Whitney U test, *p* = 0.0135). (**M**) Scatter plot of the TOP2A/CSP27 ratio versus TOP2A mRNA (FPKM) expression across all samples, with Spearman correlation (r = 0.49, 95% CI 0.13–0.74, *p* = 0.0090). *p* values in panels (**B**–**D**) were determined by two-sided Mann–Whitney U tests and adjusted for multiple comparisons using the Benjamini–Hochberg procedure within the IHC family (m = 3), and those in panels (**I**–**K**) within the FISH family (m = 3); those in panels (**F**,**L**) were determined by the Mann–Whitney U test, and that in panel (**M**) by Spearman correlation. (* *p* or q < 0.05, ** *p* or q < 0.01, **** *p* or q < 0.0001; n.s., not significant.).

To assess the concordance of TOP2A protein levels with the bulk RNA-seq findings, we performed immunohistochemistry (IHC) on 154 meningioma specimens from the same patient cohort, for which matched bulk RNA-seq data were available (114 CNS WHO Grade 1, 36 Grade 2, and 4 Grade 3). Of the 160 patients in the bulk RNA-seq cohort, the 154 with available FFPE specimens were analyzed by IHC; the IHC cohort is therefore a nested subset of the bulk cohort rather than an independent group. Representative images demonstrated markedly stronger nuclear staining intensity in high-grade tumors compared to low-grade counterparts (Figure 4A). Quantitative analysis of IHC scores revealed significantly elevated TOP2A protein levels in high-grade (Grade 2–3) tumors (Grade 1, *n* = 114 vs. Grade 2–3, *n* = 40; *p* = 5.60 × 10^−10^, Benjamini–Hochberg-adjusted q = 1.68 × 10^−9^; Figure 4B), clinically communicating subtypes (communicating, *n* = 23 vs. non-communicating, *n* = 131; *p* = 2.70 × 10^−3^, Benjamini–Hochberg-adjusted q = 2.70 × 10^−3^; Figure 4C), and recurrent cases (primary, *n* = 127 vs. recurrent, *n* = 27; *p* = 5.97 × 10^−6^, Benjamini–Hochberg-adjusted q = 8.95 × 10^−6^; Figure 4D). Kaplan–Meier analysis of 147 patients with available clinical follow-up, stratified by the optimal cutoff for TOP2A protein expression (high, score > 1, *n* = 40 vs. low, score ≤ 1, *n* = 107; Supplementary Material S7), demonstrated that high TOP2A protein levels were significantly associated with shorter progression-free survival (PFS) (log-rank *p* = 1.75 × 10^−6^, Lausen–Schumacher-corrected *p* = 9.1 × 10^−5^). Univariable Cox proportional hazards regression confirmed this association, both for high versus low TOP2A protein (HR = 8.57, 95% CI 3.00–24.51, *p* = 6.2 × 10^−5^) and for TOP2A protein as a continuous variable (HR = 1.11 per unit increase, 95% CI 1.06–1.16, *p* = 3.3 × 10^−6^) (Figure 4E). Furthermore, comparison of TOP2A mRNA levels between the protein-high and protein-low groups revealed significantly higher mRNA expression in the protein-high group (*p* = 1.6 × 10^−12^; Figure 4F). Spearman correlation analysis confirmed a positive correlation between TOP2A mRNA and protein expression levels across the matched specimens (Figure 4G; *r* = 0.65, 95% CI 0.54–0.74, *p* = 3.8 × 10^−19^).

### 2.4. FISH-Based Assessment of TOP2A Copy-Number Status

To determine whether TOP2A mRNA and protein overexpression is accompanied by focal copy-number gain, we directly quantified focal TOP2A copy number using dual-color fluorescence in situ hybridization (FISH) on 27 fresh-frozen meningioma samples with a TOP2A-specific probe and a chromosome 17 centromere (CSP27) reference probe. Representative images revealed markedly elevated TOP2A/CSP27 ratios in cases with high-level amplification (Figure 4H). Quantitative FISH analysis demonstrated significantly higher TOP2A copy-number ratios in Grade 2–3 tumors (Grade 1, *n* = 7 vs. Grade 2–3, *n* = 20; *p* = 3.35 × 10^−3^, Benjamini–Hochberg-adjusted q = 0.01005; Figure 4I). In contrast, no significant difference in TOP2A copy-number ratio was observed for clinical communication status (non-communicating, *n* = 10 vs. communicating, *n* = 17; *p* = 0.238, Benjamini–Hochberg-adjusted q = 0.238; Figure 4J) or skull base involvement (skull base, *n* = 20 vs. non-skull base, *n* = 7; *p* = 0.115, Benjamini–Hochberg-adjusted q = 0.172; Figure 4K). Furthermore, comparison of TOP2A mRNA levels between the FISH-low and FISH-high groups revealed significantly higher expression in the FISH-high group (FISH-low, TOP2A/CSP27 ratio < 1.5, *n* = 19 vs. FISH-high, ratio ≥ 1.5, *n* = 8; *p* = 0.0135, unadjusted for multiple comparisons as a confirmatory analysis; Figure 4L). Correlation analysis confirmed a positive association between the TOP2A/CSP27 ratio and TOP2A mRNA expression across the cohort (Figure 4M; r = 0.49, 95% CI 0.13–0.74, *p* = 0.0090).

## 3. Discussion

As with other malignancies, meningiomas harbor substantial intratumoral heterogeneity, a feature well documented in prior single-cell studies [7]. Emerging functional taxonomy further suggests that clinical variability across meningiomas is driven by distinct molecular states rather than by morphological heterogeneity alone [10]. Consistent with this paradigm, we observed a cell-cycle proliferation cluster with high TOP2A expression (DNA topoisomerase II alpha); because this subpopulation was defined by cell-cycle pathway enrichment, this association is not independent of the clustering criterion and is not presented as evidence of TOP2A’s prognostic priority. As a critical enzyme mediating chromosomal decatenation and segregation during the S and G2/M phases, TOP2A serves as a reliable marker of proliferative activity [11]. Extending these single-cell observations to the bulk transcriptomic level, we found that TOP2A expression was enriched in high-grade cases, and survival analysis further demonstrated that elevated TOP2A mRNA levels were significantly associated with shorter progression-free survival (PFS). These findings are consistent with previous reports identifying TOP2A/MKI67-positive subclusters as key drivers of recurrence and aggressive behavior in atypical meningiomas [8,12]. Notably, while Fan et al. [8] first described this proliferative neoplastic subpopulation at the single-cell level in a five-case cohort, that study did not validate TOP2A focal copy-number status, did not confirm protein overexpression in a large clinical cohort, and did not address its therapeutic implications. The present study extends these observations by characterizing TOP2A at the mRNA, protein, and copy-number levels in a substantially larger clinical cohort.

The cross-platform concordance of TOP2A—mRNA and protein overexpression together with focal copy-number gain—supports its candidacy as a candidate biomarker rather than merely a transcriptional correlate of proliferation. We note that BIRC5, the other candidate detected in every sample, showed an essentially equivalent univariate association with PFS (standardised per-SD HR 2.25 vs. 2.27 for TOP2A); TOP2A is therefore not distinguished from BIRC5 by effect size and was advanced on the basis of its druggability and the availability of validated IHC and FISH assays. Relative to Ki-67/MIB-1, the routine proliferation marker in meningioma, TOP2A added statistically significant prognostic information when both markers were modelled together (likelihood-ratio *p* = 7.7 × 10^−3^), although discrimination was not further improved (ΔC-index ≈ 0). TOP2A should therefore be regarded not as a replacement for Ki-67 but as a complementary proliferation-associated marker that additionally offers a druggable target and a copy-number dimension. While TOP2A overexpression has been documented in aggressive meningiomas [11], our data show that focal copy-number gain is enriched in high-grade cases, paralleling the expression pattern. Notably, TOP2A mRNA and protein overexpression were each associated with shorter PFS, offering a stratification axis beyond histopathology alone [13]. Furthermore, TOP2A amplification has been shown to predict enhanced chemosensitivity in HER2-positive breast cancer [14,15] and to define a subset of patients who derive substantial benefit from anthracycline-based regimens [16]. More recently, elevated TOP2A expression has been leveraged to rationalize combination strategies—such as etoposide combined with EZH2 inhibition—in aggressive prostate cancer [17], extending its therapeutic relevance beyond conventional cytotoxic regimens. In meningioma, prior work established TOP2A protein overexpression as a superior predictor of recurrence compared with MIB-1 labeling [11,18]. A recent large-scale multi-omic study [10] has comprehensively characterized meningioma cell states and their association with progression and outcome; however, that work did not specifically examine TOP2A copy-number amplification or its candidate-biomarker dimension. Our study extends these findings by demonstrating focal TOP2A copy-number gain in high-grade cases and supporting its potential as a candidate prognostic biomarker. Building on these findings, prospective clinical studies are warranted to evaluate whether TOP2A copy-number status can guide risk-adapted treatment strategies and identify patients most likely to benefit from TOP2A-directed therapies in meningioma.

While our integrated multi-omic approach provides a high-resolution atlas of meningioma heterogeneity, several limitations warrant consideration. The single-cell component of this study was based on only two recurrent, higher-grade tumors, and conclusions derived from it—the six functional subpopulations identified by GSVA and the cell-cycle proliferation cluster marked by TOP2A—should therefore be regarded as exploratory and hypothesis-generating rather than definitive. These findings may not be generalizable to the full spectrum of meningioma biology, particularly benign or primary tumors, and will require replication in larger single-cell cohorts before they inform clinical decisions. The number of Grade 3 tumors was also small (*n* = 5 of 160 in the bulk RNA-seq cohort and *n* = 4 of 154 in the IHC cohort); Grade 2 and Grade 3 were therefore analyzed together as a single higher-grade group, and conclusions specific to Grade 3 should be interpreted with caution. The prognostic analyses were also constrained by the small number of progression events (19 of 154 patients, 12.3%), which limits the number of covariates that can be reliably modelled and widens the confidence intervals of the fully adjusted models; these findings therefore require confirmation in larger cohorts with longer follow-up. Future investigations should prioritize expanding single-cell profiling to larger, treatment-naïve cohorts and incorporating spatial technologies to map the topological relationships between proliferative clones and the microenvironment. Addressing these gaps will be crucial to translating these molecular insights into clinical trials.

## 4. Materials and Methods

### 4.1. Study Participants and Design

The study protocol was approved by the Ethics Committee of the National Cancer Center/Cancer Hospital, Chinese Academy of Medical Sciences (approval number: 26/131-5855 (NCC6037)), and conducted in accordance with the Declaration of Helsinki and ICH-GCP [19,20], with written informed consent obtained from all participants before enrollment. This retrospective cohort included 162 patients with a clinical diagnosis of meningioma who underwent treatment at the same institution between 2014 and 2021. To standardize histopathologic classification and grading across the entire study period, all diagnoses were centrally re-reviewed and aligned with the 2024 edition of the National Comprehensive Cancer Network (NCCN) Clinical Practice Guidelines in Oncology (NCCN Guidelines^®^) for meningioma management [21]. To ensure analytical validity, the following rigorous exclusion criteria were applied: ambiguous or non-meningioma pathology; unavailable/archived tissue (insufficient quantity, improper storage, or no residual material); preoperative radiotherapy or systemic therapy; coexisting second malignancy; refusal or loss to follow-up; and samples with extensive tumor necrosis, calcification, or fibrosis liable to compromise molecular/histologic assessment.

Meningiomas were classified according to two independent criteria. First, by tumor location: tumors arising from the skull-base dural surfaces (anterior, middle, or posterior cranial fossa) were classified as skull base, and all others as non-skull base. Second, by growth pattern: tumors were dichotomized as communicating versus non-communicating. A communicating meningioma was defined as a meningioma originating from the intracranial meninges that extended contiguously through a natural anatomical channel (e.g., the optic canal, superior orbital fissure, or jugular foramen), a cranial suture or neurovascular foramen, or an area of bone erosion/destruction into an adjacent extracranial compartment—including the orbit, nasal cavity and paranasal sinuses, pterygopalatine or infratemporal fossa, or the neck—thereby forming a single continuous intra-extracranial lesion. Non-communicating meningiomas remained confined to the intracranial compartment. Classification was based on preoperative magnetic resonance imaging (and/or computed tomography) and confirmed by intraoperative findings.

Meningioma specimens without significant hemorrhage, necrosis, or thermal damage were collected intraoperatively. Samples designated for single-cell isolation were immediately placed in MACS Tissue Storage Solution (Miltenyi Biotec). All samples were maintained at 4 °C until further processing, with minimization of cold ischemia time deemed critical for maximizing experimental success. A total of 162 meningioma samples were enrolled; single-cell RNA sequencing (scRNA-seq) was performed on tumor tissues from two representative cases, and validation utilized bulk mRNA sequencing data generated from 160 of the enrolled cases.

Clinical parameters collected in this study encompassed demographic characteristics, laboratory test results, and relevant clinical information. All terminology abbreviations, calculation formulas, and clinical data were standardized in accordance with international standards.

### 4.2. Single-Cell RNA Sequencing and Analysis

scRNA-seq was performed using the 10× Chromium Single Cell 3′ v2 reagent kit (10× Genomics, Pleasanton, CA, USA) according to the manufacturer’s protocol. The target number of cells captured per sample ranged from 2000 to 10,000. Sequencing libraries were constructed following the manufacturer’s instructions and sequenced on an Illumina NovaSeq 6000 platform (Illumina, San Diego, CA, USA). Raw sequencing data were processed through the Cell Ranger pipeline (version 2.1.0; 10× Genomics, Pleasanton, CA, USA). Reads were aligned to the human reference genome (hg19/GRCh37), and gene expression counts were quantified to generate a unique molecular identifier (UMI)–based matrix indexed by cell barcodes.

Quality control (QC) and preprocessing were performed on the scRNA-seq data prior to downstream analysis. Low-quality cells were filtered out based on the following criteria: detection in fewer than three cells, or expression of fewer than 300 genes. Subsequently, data from the remaining high-quality (core) cells were integrated using the Seurat R package (version 5.4.0; Bioconductor, Buffalo, NY, USA) via an anchor-based integration method. Gene expression was normalized using a linear regression model. To mitigate the impact of technical artifacts arising from two or more cells sequenced under a single barcode (doublets), we employed the DoubletFinder R package (version 2.0.6; Bioconductor, Buffalo, NY, USA). Thereafter, the top 2000 highly variable genes (HVGs) were identified utilizing the “vst” method. Principal component analysis (PCA) was performed on the normalized data, and the top 20 principal components (PCs) were selected for downstream analysis. Dimensionality reduction was visualized using the Uniform Manifold Approximation and Projection (UMAP) algorithm based on these 20 PCs. For cell type identification, the SingleR package (Bioconductor, Buffalo, NY, USA) was utilized to perform initial automated annotation, referencing canonical datasets including HumanPrimaryCellAtlasData, BlueprintEncodeData, and ImmuneCellExpressionData. To refine these annotations, differentially expressed marker genes for each cluster were identified using the FindAllMarkers function in Seurat. Subsequent manual curation was guided by known marker genes retrieved from the CellMarker database (http://117.50.127.228/CellMarker/ (accessed on 15 March 2026)) and relevant literature. Quality control criteria were applied consistently across both samples using biologically informed thresholds: cells were filtered out if they exhibited (1) gene counts ≤ 200 or Unique Molecular Identifier (UMI) counts ≤ 1000, targeting low-quality cells and empty droplets; (2) gene counts ≥ 10,000 or UMI counts ≥ 100,000, reducing potential doublet artifacts; or (3) mitochondrial gene proportions ≥ 20%. DoubletFinder analysis estimated an overall doublet rate of 15.4%.

To stratify the malignant compartment into functionally discrete subpopulations, Gene Set Variation Analysis (GSVA) was performed using the GSVA R package (version 1.45; Bioconductor, Buffalo, NY, USA) on the tumor-cell expression matrix. GSVA computes a pathway-enrichment score for each predefined gene set in every cell, operating at the gene-set rather than the single-gene level. Cells were then clustered on the basis of these pathway-enrichment scores, yielding seven primary clusters (g0, g3, g4, g8, g11, g5 and g7); because clusters g3 and g4 lay closest to each other in the UMAP embedding, they were combined into a single population, yielding six functionally discrete subpopulations: Cell cycle proliferation, MYC-driven proliferation, Angiogenesis, Mesenchymal activation program, MYC-targets, and Lipid metabolism (Supplementary Figure S3).

Marker genes characterizing each functional subpopulation were identified using the FindAllMarkers function in Seurat, which performs a single-gene Wilcoxon rank-sum test on per-gene expression values and ranks genes by average log2 fold change (avg_log_2_FC). Because the functional subpopulations were defined by pathway-enrichment scores, markers of a given subpopulation are by construction enriched for genes of the corresponding pathway; the top-ranked markers of the cell-cycle subpopulation were therefore not used as a criterion for prioritising candidate genes. The top-50 cutoff for candidate-marker screening follows the practice adopted in previous single-cell studies of meningioma, in which downstream comparisons were restricted to the highest-ranked markers of each subpopulation [7,8,10].

### 4.3. Identification of Malignant Cells

Single-cell copy number variation (CNV) analysis was performed using inferCNV 1.20.0^25^ (https://github.com/broadinstitute/inferCNV (accessed on 15 March 2026); Broad Institute, Cambridge, MA, USA). A raw gene expression counts matrix, cell annotation file, and gene–chromosome position file were formatted according to the tool’s specifications. Given the absence of a distinct normal cell population for baseline subtraction, analyses were conducted in reference-free mode, wherein a genome-wide baseline was computationally derived from the entire cohort. A gene sliding window approach combined with Pyramid Smoothing was applied to normalize read depth across the genome. To minimize technical noise, genes with an average read count < 0.1 across all cells were excluded prior to analysis. Furthermore, both the observed (tumor) cell datasets and the computational baseline were randomly downsampled to a uniform depth to balance computational efficiency and sensitivity.

### 4.4. Bulk RNA Sequencing and Analysis

Total RNA was extracted from fresh-frozen meningioma tissues (*n* = 160) using the RNeasy Plus Mini Kit (Qiagen, Hilden, Germany), following the manufacturer’s protocol. RNA quality and quantity were assessed using an Agilent Bioanalyzer 2100 (Agilent Technologies, Santa Clara, CA, USA), with only samples exhibiting an RNA Integrity Number (RIN) ≥ 7.0 retained for library construction. Sequencing libraries were prepared using the NEBNext Ultra II Directional RNA Library Prep Kit for Illumina (New England Biolabs, Ipswich, MA, USA) with poly(A) selection. Libraries were sequenced on an Illumina NovaSeq 6000 platform (Illumina, San Diego, CA, USA) with paired-end 150-bp reads to achieve a minimum depth of 100 million clean reads per sample.

Raw sequencing data (FASTQ format) were processed through a standardized pipeline. Quality control was performed usingFastQC (v0.11.9; Babraham Institute, Cambridge, UK) and MultiQC (v1.12; Babraham Institute, Cambridge, UK). Adapter trimming and quality filtering were conducted with Trim Galore (v0.6.7; Babraham Institute, Cambridge, UK). Clean reads were aligned to the human reference genome (GRCh38/hg38) using the STAR aligner (v2.7.10a; Cold Spring Harbor Laboratory, Cold Spring Harbor, NY, USA). Gene-level quantification was performed using featureCounts (v2.0.3; Subread package, Bioconductor, Buffalo, NY, USA). Expression levels were normalized and reported as fragments per kilobase of transcript per million mapped reads (FPKM). Differential expression analysis between Grade 1 and Grade 2–3 tumors was performed using the DESeq2 R package (v1.40.2; Bioconductor, Buffalo, NY, USA), which fits a negative-binomial generalized linear model with a Wald test; genes were called significant at a false discovery rate (FDR) < 0.001 and |log_2_FC| > 2.0. Sequencing quality metrics for all 160 bulk RNA-seq libraries are provided in the Supplementary Materials. Briefly, an average of 119.3 million clean reads were obtained per sample, with a mean Q30 of 92.1% and a mean uniquely mapped read rate of 81.6% as determined by STAR.

### 4.5. Immunohistochemistry (IHC) Staining

To assess TOP2A protein expression, immunohistochemistry was performed on 154 formalin-fixed, paraffin-embedded (FFPE) meningioma specimens from the same patient cohort, for which matched bulk RNA-seq data were available. Briefly, 4-μm tissue sections were deparaffinized in xylene, rehydrated through graded ethanol, and subjected to antigen retrieval using citrate buffer (pH 6.0) in a pressure cooker. Endogenous peroxidase activity was blocked with 3% hydrogen peroxide for 10 min. Sections were incubated overnight at 4 °C with a rabbit anti-TOP2A primary antibody (dilution 1:200; Proteintech, Wuhan, China). Visualization was achieved usingdiaminobenzidine (DAB) substrate (Dako, Glostrup, Denmark), and nuclei were counterstained with hematoxylin (Sigma-Aldrich, St. Louis, MO, USA). IHC scoring was independently performed by two board-certified pathologists blinded to clinical data. Scoring used a semi-quantitative H-score that combines the intensity of nuclear staining with the proportion of TOP2A-positive tumor cells; for survival analysis, scores were dichotomized at the cutoff that maximized the log-rank statistic (high, >1 vs. low, ≤1).

### 4.6. Fluorescence In Situ Hybridization (FISH)

Dual-color FISH was conducted on 27 fresh-frozen meningioma samples to assess TOP2A copy-number status. Briefly, 5-μm frozen sections were fixed in methanol/acetic acid (3:1), dehydrated in ethanol series (70%, 85%, 100%), and hybridized with a TOP2A-specific probe (labeled with SpectrumOrange) and a chromosome 17 centromere (CSP27) reference probe (labeled with SpectrumGreen; Abbott Molecular, Des Plaines, IL, USA) according to the manufacturer’s protocol. Post-hybridization washes were performed with 0.4× SSC/0.3% NP-40 at 72 °C for 2 min. Signals were enumerated in 200 non-overlapping tumor cell nuclei per sample under a fluorescence microscope (Olympus BX53; Olympus, Tokyo, Japan). The TOP2A/CSP27 ratio was calculated as the mean number of TOP2A signals divided by the mean number of CSP27 signals per nucleus. Following standard dual-probe conventions, ratio ≥ 2.0 was defined as high-level TOP2A amplification [22], whereas ratio 1.5–1.99 was classified as low-level copy number gain, and ratio < 1.5 as diploid status. Because high-level amplification was present in only two of the 27 samples, the FISH-high group used for the two descriptive comparisons of TOP2A mRNA reported below (Figure 4L) and for the correlation analysis shown in Figure 4M was defined as a TOP2A/CSP27 ratio ≥ 1.5 (*n* = 8), with the remaining samples (ratio < 1.5, *n* = 19) designated FISH-low.

### 4.7. The Univariate Cox Proportional Hazards Regression of 11 Candidate Genes

The prognostic significance of gene expression was evaluated by univariate Cox proportional hazards regression, fitted separately for each of the 11 candidate genes. Gene expression levels were log_2_(x + 1)-transformed and modeled as continuous variables; accordingly, the hazard ratio (HR) and its 95% confidence interval (CI) reflect the change in the hazard of progression per doubling (2-fold increase) of expression. *p* values were derived from the Wald test, and multiple testing across the 11 genes was controlled using the Benjamini–Hochberg procedure to obtain false discovery rate (FDR)-adjusted q values. A two-sided *p* < 0.05 was considered statistically significant. These univariate Cox regression analyses were performed using the lifelines package (version 0.30.3) in Python (version 3.13).

### 4.8. Cox Proportional Hazards Regression Analysis of Prognostic Factors

Cox proportional hazards regression was performed using Python (version 3.13) with the lifelines package. Progression-free survival (PFS) was the primary endpoint. Univariate Cox proportional hazards regression was first applied to each candidate variable, and variables with *p* < 0.05 were advanced to the multivariate model. Multicollinearity was evaluated using the variance inflation factor (VIF). A multivariate Cox regression model was then built by forward selection with TOP2A mRNA expression forced in as the primary exposure; ordered variables (WHO grade and Simpson grade) were modeled as linear trends, and Ki-67 was scaled per 10% increment. To assess the robustness of the final model, forward selection without forcing TOP2A and backward elimination (removal *p* ≥ 0.05) were additionally performed, and all three variable-selection strategies converged on the identical model. Chemotherapy was excluded from the model owing to complete separation (only two treated patients). Effect sizes are reported as hazard ratios (HRs) with 95% confidence intervals (CIs). Model discrimination was assessed by the concordance index (C-index), and the overall model was tested by the likelihood ratio and Wald tests. The proportional hazards assumption was verified using scaled Schoenfeld residuals. Two sensitivity analyses were performed. First, to test whether TOP2A adds prognostic information beyond Ki-67, a model containing both markers was fitted and compared with the corresponding model without TOP2A by likelihood-ratio test, AIC and C-index. Second, a fully adjusted model was fitted in which TOP2A mRNA, WHO grade, Simpson grade, age, adjuvant radiotherapy and Ki-67 were all forced in irrespective of their univariate *p* value; given the limited number of progression events (19 events), this model was additionally evaluated by leave-one-covariate-out refitting and by bootstrap resampling. All tests were two-sided, and *p* < 0.05 was considered statistically significant.

### 4.9. Statistical Analysis

Bioinformatic analyses of the single-cell and bulk transcriptomic data were performed using R software (version 4.4.3; R Foundation for Statistical Computing, Vienna, Austria); group comparisons and Cox proportional hazards regression were performed in Python (version 3.13; Python Software Foundation, Beaverton, OR, USA) using the lifelines (version 0.30.3; PyPI, https://pypi.org/project/lifelines/ (accessed on 15 March 2026)) and SciPy (version 1.13.1; SciPy developers, https://scipy.org) packages. Continuous variables were assessed for normality prior to analysis. Normally distributed data are presented as the mean ± standard deviation (SD) and were compared using independent-sample *t*-tests. Non-normally distributed continuous variables are expressed as the median (interquartile range) and were compared using the Mann–Whitney U test. Categorical variables were summarized as frequencies and percentages, with intergroup differences evaluated using the chi-square test or Fisher’s exact test, as appropriate. Multiple testing was controlled within assay-platform families rather than across all comparisons. The ten intergroup comparisons of TOP2A expression were grouped by platform: bulk RNA-seq (m = 4; Figure 3F–I), IHC (m = 3; Figure 4B–D) and FISH (m = 3; Figure 4I–K). Two-sided Mann–Whitney U tests were performed using the asymptotic normal approximation with tie and continuity corrections, and within each family the resulting *p* values were adjusted using the Benjamini–Hochberg procedure to obtain false discovery rate (FDR)-adjusted q values. Both unadjusted *p* values and Benjamini–Hochberg-adjusted q values are reported, and significance for these comparisons was assigned only when q < 0.05, whereas comparisons with a nominal *p* < 0.05 that did not retain significance after correction are described as trends. Holm step-down and Bonferroni corrections were additionally performed and yielded concordant conclusions. Two further comparisons (Figure 4F,L) fall outside these families and are reported unadjusted as confirmatory analyses. Multiple testing corrections were otherwise applied where indicated, as in the univariate Cox analysis of the 11 candidate genes. Kaplan–Meier analysis was conducted using GraphPad Prism (version 10.0; GraphPad Software, Boston, MA, USA). Median survival time was estimated via the Kaplan–Meier method, with differences between survival curves compared via the log-rank test. For dichotomized survival analyses, patients were stratified into high- and low-expression (or protein) groups at the cutoff that maximized the log-rank statistic (maximally selected rank statistic; candidate cutoffs were restricted to those leaving at least 10% of the cohort in each group); because selecting the cutoff from the same dataset inflates the type I error, the log-rank *p* values were additionally corrected for cutoff selection using the Lausen–Schumacher method, and both uncorrected and corrected *p* values are reported. A two-tailed *p* < 0.05 was considered statistically significant.

## 5. Conclusions

In summary, this study integrates single-cell and bulk transcriptomics with multi-level experimental validation to explore the molecular architecture of meningioma. In an exploratory single-cell cohort of two recurrent, higher-grade cases, we observed a TOP2A-high proliferative subpopulation—consistent with prior single-cell reports; these observations therefore remain hypothesis-generating. The central finding is that TOP2A mRNA and protein overexpression is associated with shorter progression-free survival, whereas focal TOP2A copy-number gain, detected by FISH, is enriched in high-grade cases and concordant with TOP2A expression; the limited size of the FISH subset, however, did not permit an independent survival analysis. While preliminary, derived from a two-patient exploratory single-cell cohort alongside a larger bulk validation cohort, and requiring prospective validation, these findings support the rationale for TOP2A-guided risk stratification and TOP2A-directed therapeutic strategies in high-grade meningioma.

## Figures and Tables

**Table 1:** Univariate Cox proportional hazards regression analysis of progression-free survival.

Variable	HR	95% CI	*p* Value
WHO grade (per grade)	4.24	2.31–7.78	3.1 × 10^−6^
Presentation (recurrent vs. primary)	17.65	6.21–50.17	7.3 × 10^−8^
Location (skull base vs. non-skull base)	4.72	1.37–16.31	0.014
Communicating (yes vs. no)	6.39	2.53–16.14	8.6 × 10^−5^
No. of tissue blocks (per 1)	1.42	1.18–1.71	2.5 × 10^−4^
Sex (male vs. female)	2.60	1.03–6.57	0.043
Age (per year)	0.99	0.96–1.03	0.724
Simpson grade (per grade)	2.09	1.38–3.17	5.4 × 10^−4^
Complication (yes vs. no)	1.49	0.53–4.20	0.454
Radiotherapy (yes vs. no)	2.75	0.98–7.72	0.055
Ki-67 (per 10%)	2.25	1.57–3.22	9.6 × 10^−6^
TOP2A mRNA (per log_2_ unit)	1.76	1.38–2.25	5.6 × 10^−6^

Note: HR, hazard ratio; CI, confidence interval. Univariate Cox regression was performed separately for each candidate variable. HR > 1 indicates an increased risk of progression. The outcome was progression-free survival (PFS) in months, with status as the event indicator (1 = progression, 0 = censored). *p* values are from the Wald test.

**Table 2:** Multivariate Cox proportional hazards regression analysis of progression-free survival.

Variable	HR	95% CI	*p* Value
TOP2A mRNA (per log_2_ unit)	1.54	1.20–1.98	6.4 × 10^−4^
Presentation (recurrent vs. primary)	10.26	3.29–32.02	6.0 × 10^−5^
Simpson grade (per grade)	1.98	1.22–3.21	0.006

Note: HR, hazard ratio; CI, confidence interval. The final multivariate model was derived by forward selection (entry *p* < 0.05) with TOP2A mRNA forced into the model as the primary exposure. Covariates retained: TOP2A mRNA (per log_2_ unit), presentation (recurrent vs. primary), and Simpson grade (per grade). *p* values are from the Wald test. Among the 154 patients analysed, 19 progression events (12.3%) were observed during follow-up.

## Data Availability

The data that support the findings of this study are available from the corresponding author or Hongqing Cai (phonecy@126.com) upon reasonable request.

## Supplementary Materials

The following supporting information can be downloaded at: https://www.mdpi.com/article/10.3390/ijms27208984/s1.
