# Supplementary material for: TOP2A Overexpression as a Prognostic Biomarker in High-Grade Meningioma: An Integrated Single-Cell and Bulk Transcriptomic Study
Source: Int J Mol Sci. 2026 Oct 10;27(20):8984. doi: 10.3390/ijms27208984 (PMC13704434; doi:10.3390/ijms27208984)
Supplement: Supplementary file 1 [file ijms-27-08984-s001.zip › Supplementary Table S10_TOP2A_incremental_value_beyond_Ki67.pdf]

Supplementary Table S10. Incremental prognostic value of TOP2A beyond Ki-67: nested multivariable Cox models

Progression-free survival, n = 154 patients; 19 progression events (12.3%).

(A) Nested models

Model	Covariate	HR (95% CI)	P value	AIC	C-index
M1	Presentation (recurrent vs primary)	14.53 (4.96–42.58)	1.1×10^{-6}	138.15	0.862
M1	Simpson grade (per grade)	1.51 (1.00–2.28)	0.049		
M2	Presentation (recurrent vs primary)	10.67 (3.43–33.21)	4.4×10^{-5}	134.73	0.899
M2	Simpson grade (per grade)	1.73 (1.09–2.73)	0.020		
M2	Ki-67 (per 10%)	1.67 (1.13–2.47)	0.010		
M3	Presentation (recurrent vs primary)	9.42 (2.98–29.76)	1.3×10^{-4}	129.63	0.897
M3	Simpson grade (per grade)	2.05 (1.24–3.37)	0.005		
M3	Ki-67 (per 10%)	1.41 (0.82–2.46)	0.218		
M3	TOP2A mRNA (per $\log_2$ unit)	1.42 (1.08–1.86)	0.011		

M1 = presentation status + Simpson grade (clinical base model); M2 = M1 + Ki-67; M3 = M2 + TOP2A mRNA.

(B) Nested likelihood-ratio comparisons

Comparison	Likelihood-ratio χ^2	df	P value	ΔAIC	ΔC-index
M2 vs M1 (adding Ki-67)	5.42	1	0.020	−3.42	+0.036
M3 vs M2 (adding TOP2A to a model already containing Ki-67)	7.10	1	7.7×10^{-3}	−5.10	−0.002
M3 vs M1 (adding Ki-67 and TOP2A together)	12.52	2	1.9×10^{-3}	−8.52	+0.035

Note: Adding TOP2A to a model that already contained Ki-67 produced a statistically significant improvement in model fit (M3 vs M2: likelihood-ratio test $\chi^2 = 7.10$, df = 1, $P = 7.7 \times 10^{-3}$; ΔAIC = −5.10 favouring M3; continuous net reclassification improvement = +0.79), whereas discrimination was not further improved (ΔC-index = −0.002; out-of-bag bootstrap, 507 evaluable replicates: median ΔC-index = −0.005, 95% CI −0.106 to +0.065). In the joint model Ki-67 no longer reached significance (P = 0.218) while TOP2A remained significant (P = 0.011). Taken together, these results indicate that TOP2A carries prognostic information beyond Ki-67, but that the two proliferation markers capture largely overlapping information, so that TOP2A is best regarded as a complementary rather than a superior or additive marker. HR, hazard ratio; CI, confidence interval; AIC, Akaike information criterion; C-index, concordance index. P values are from the Wald test (coefficients) and the likelihood-ratio test (model comparisons).
