# Supplementary material for: TOP2A Overexpression as a Prognostic Biomarker in High-Grade Meningioma: An Integrated Single-Cell and Bulk Transcriptomic Study
Source: Int J Mol Sci. 2026 Oct 10;27(20):8984. doi: 10.3390/ijms27208984 (PMC13704434; doi:10.3390/ijms27208984)
Supplement: Supplementary file 1 [file ijms-27-08984-s001.zip › Supplementary Table S1_Clinical characteristics of two patients profiled by scRNA-seq.pdf]

**Supplementary Table S1. Clinical characteristics of two meningioma patients profiled by single-cell sequencing**

| Clinical features | Case1 | Case2 |
|---|---|---|
| Gender | Female | Female |
| Age | 61 | 68 |
| Tumor status | Recurrent | Recurrent |
| Pathological diagnosis | Atypical meningioma | Atypical meningioma |
| Lesion location | Left frontal parasagittal (anterior third) | Right anteromedial skull base |
| Lesion size (mm) | 61*54*49 | 46*32*45 |
| En Plaque meningioma (Yes/No) | No | Yes |
| WHO grade | 2 | 3 |
| Treatment protocol | Resection | Resection + Radiotherapy |
| Simpson grade | 1 | 2 |
| Follow-up outcome | Recurrent | Recurrent |
| Follow-up duration (Months) | 25 | 19 |

WHO Grade, World Health Organization Histological Grade.
