# Supplementary material for: TOP2A Overexpression as a Prognostic Biomarker in High-Grade Meningioma: An Integrated Single-Cell and Bulk Transcriptomic Study
Source: Int J Mol Sci. 2026 Oct 10;27(20):8984. doi: 10.3390/ijms27208984 (PMC13704434; doi:10.3390/ijms27208984)
Supplement: Supplementary file 1 [file ijms-27-08984-s001.zip › Supplementary Table S2_Univariate Cox of 11 candidate genes.pdf]

Supplementary Table S2. Univariate Cox proportional hazards regression of 11 candidate genes for progression-free survival in meningioma patients (N = 154)

Gene	HR (95% CI)	P value	FDR q	HR per SD (95% CI)
LINC01524*†	1.779 (1.365–2.318)	1.98×10^{-5}	4.36×10^{-5}	1.661 (1.316–2.097)
TOP2A*†	1.763 (1.380–2.252)	5.58×10^{-6}	2.04×10^{-5}	2.270 (1.594–3.234)
BIRC5*†	1.738 (1.353–2.234)	1.56×10^{-5}	4.28×10^{-5}	2.246 (1.556–3.241)
FMO3*†	1.447 (1.165–1.797)	8.44×10^{-4}	0.001	1.861 (1.292–2.679)
CHP2*†	1.368 (1.214–1.542)	2.74×10^{-7}	3.01×10^{-6}	1.878 (1.477–2.388)
SLC6A1*†	1.344 (1.145–1.578)	2.98×10^{-4}	5.46×10^{-4}	1.872 (1.333–2.630)
C5orf66-AS1*†	1.338 (1.186–1.510)	2.13×10^{-6}	1.17×10^{-5}	1.649 (1.341–2.028)
RBFOX1*†	1.323 (1.104–1.586)	0.002	0.002	1.720 (1.211–2.444)
NLGN4X*†	1.302 (1.103–1.538)	0.002	0.002	1.792 (1.241–2.587)
SHISA9*†	1.283 (1.118–1.473)	4.00×10^{-4}	6.29×10^{-4}	1.719 (1.274–2.321)
NNAT*†	1.206 (1.070–1.358)	0.002	0.002	1.728 (1.219–2.449)

HR, hazard ratio; CI, confidence interval; PFS, progression-free survival; FDR, false discovery rate.

Gene expression was log2(x + 1)-transformed and modeled as a continuous variable; HR denotes the hazard of progression/recurrence per one-fold increase in expression. The rightmost column reports standardised hazard ratios per one

standard deviation (SD) of log2(x + 1)-transformed expression, which places all 11 genes on a common scale and allows their effect sizes to be compared directly regardless of each gene's variance.

P values were derived from the Wald test; FDR q values were adjusted for 11 tests using the Benjamini–Hochberg method.

*P < 0.05; †FDR q < 0.05. Among 154 patients, 19 (12.3%) progression events were observed; no missing values were present.
