# Supplementary material for: TOP2A Overexpression as a Prognostic Biomarker in High-Grade Meningioma: An Integrated Single-Cell and Bulk Transcriptomic Study
Source: Int J Mol Sci. 2026 Oct 10;27(20):8984. doi: 10.3390/ijms27208984 (PMC13704434; doi:10.3390/ijms27208984)
Supplement: Supplementary file 1 [file ijms-27-08984-s001.zip › Supplementary Table S3_Model_Fit_Tests.pdf]

## Supplementary Table S3. Global model fit and significance tests of the final multivariate Cox model

| Test | Statistic | df | P value |
|---|---|---|---|
| Likelihood ratio test | 47.57 | 3 | <0.001 |
| Wald test (global) | 40.40 | 3 | <0.001 |
| C-index (concordance) | 0.887 | | |
| AIC | 128.88 | | |

Note: df, degrees of freedom; AIC, Akaike information criterion; C-index, concordance index. The likelihood ratio and Wald tests assess the overall significance of the model. The C-index measures discrimination and ranges from 0.5 (no discrimination) to 1.0 (perfect discrimination).
