# Supplementary material for: TOP2A Overexpression as a Prognostic Biomarker in High-Grade Meningioma: An Integrated Single-Cell and Bulk Transcriptomic Study
Source: Int J Mol Sci. 2026 Oct 10;27(20):8984. doi: 10.3390/ijms27208984 (PMC13704434; doi:10.3390/ijms27208984)
Supplement: Supplementary file 1 [file ijms-27-08984-s001.zip › Supplementary Table S4_VIF_Collinearity.pdf]

Supplementary Table S4. Variance inflation factor (VIF) collinearity diagnostics

Variable	VIF
WHO grade (per grade)	2.27
Ki-67 (per 10%)	2.07
Presentation (recurrent vs primary)	1.76
Communicating (yes vs no)	1.69
TOP2A mRNA (per log2 unit)	1.59
Radiotherapy (yes vs no)	1.54
Location (skull base vs non-skull base)	1.29
Sex (male vs female)	1.22
Complication (yes vs no)	1.20
Simpson grade (per grade)	1.18
No. of tissue blocks (per 1)	1.18
Age (per year)	1.08

Note: VIF, variance inflation factor. Each candidate predictor was regressed on all other predictors to compute its VIF. VIF < 5 indicates no problematic multicollinearity; all variables showed VIF < 2.3.
