# Supplementary material for: TOP2A Overexpression as a Prognostic Biomarker in High-Grade Meningioma: An Integrated Single-Cell and Bulk Transcriptomic Study
Source: Int J Mol Sci. 2026 Oct 10;27(20):8984. doi: 10.3390/ijms27208984 (PMC13704434; doi:10.3390/ijms27208984)
Supplement: Supplementary file 1 [file ijms-27-08984-s001.zip › Supplementary Table S5_TOP2A_Adjustment_Ladder.pdf]

Supplementary Table S5. Association of TOP2A mRNA with progression-free survival across sequential adjustment models

Model	TOP2A HR	95% CI	P value
Crude (TOP2A only)	1.76	1.38–2.25	<0.001
Adjusted for WHO grade	1.44	1.07–1.94	0.015
+ presentation	1.34	1.04–1.72	0.023
+ Simpson grade (fully adjusted)	1.47	1.13–1.93	0.005

Note: HR, hazard ratio; CI, confidence interval. Each row reports the HR for TOP2A mRNA (per log2 unit) after progressively adding covariates, from the crude model to the fully adjusted model (WHO grade + presentation + Simpson grade).
