# Supplementary material for: TOP2A Overexpression as a Prognostic Biomarker in High-Grade Meningioma: An Integrated Single-Cell and Bulk Transcriptomic Study
Source: Int J Mol Sci. 2026 Oct 10;27(20):8984. doi: 10.3390/ijms27208984 (PMC13704434; doi:10.3390/ijms27208984)
Supplement: Supplementary file 1 [file ijms-27-08984-s001.zip › Supplementary Table S6_Sensitivity_Ki67.pdf]

Supplementary Table S6. Sensitivity analysis: multivariate model with Ki-67 replacing TOP2A mRNA

Variable	HR	95% CI	P value
Ki-67 (per 10%)	1.67	1.13–2.47	0.010
Presentation (recurrent vs primary)	10.67	3.43–33.21	<0.001
Simpson grade (per grade)	1.73	1.09–2.73	0.020

Note: HR, hazard ratio; CI, confidence interval. Ki-67 (per 10%) was substituted for TOP2A mRNA to test the robustness of the main findings; the model otherwise retained the same covariates (presentation, Simpson grade). P values are from the Wald test.
