# Supplementary material for: TOP2A Overexpression as a Prognostic Biomarker in High-Grade Meningioma: An Integrated Single-Cell and Bulk Transcriptomic Study
Source: Int J Mol Sci. 2026 Oct 10;27(20):8984. doi: 10.3390/ijms27208984 (PMC13704434; doi:10.3390/ijms27208984)
Supplement: Supplementary file 1 [file ijms-27-08984-s001.zip › Supplementary Table S7_PH_Assumption_Test.pdf]

Supplementary Table S7. Proportional hazards (PH) assumption test based on scaled Schoenfeld residuals

**Variable**	**rho**	**P value**
TOP2A mRNA (per log2 unit)	0.363	0.127
Presentation (recurrent vs primary)	-0.116	0.636
Simpson grade (per grade)	-0.326	0.173

Note: rho, correlation between scaled Schoenfeld residuals and time. A non-significant P value ($P > 0.05$) indicates that the proportional hazards assumption is not violated for that variable.
