# Supplementary material for: TOP2A Overexpression as a Prognostic Biomarker in High-Grade Meningioma: An Integrated Single-Cell and Bulk Transcriptomic Study
Source: Int J Mol Sci. 2026 Oct 10;27(20):8984. doi: 10.3390/ijms27208984 (PMC13704434; doi:10.3390/ijms27208984)
Supplement: Supplementary file 1 [file ijms-27-08984-s001.zip › Supplementary Table S8_Model_Comparison.pdf]

Supplementary Table S8. Robustness of TOP2A mRNA as an independent prognostic factor for progression-free survival across variable-selection strategies

Strategy	Selected variables	TOP2A HR (95% CI)	TOP2A P value
Forward selection (TOP2A forced)	TOP2A + presentation + Simpson grade	1.54 (1.20–1.98)	<0.001
Forward selection (TOP2A not forced)	presentation + TOP2A + Simpson grade	1.54 (1.20–1.98)	<0.001
Backward elimination (TOP2A not forced)	presentation + Simpson grade + TOP2A	1.54 (1.20–1.98)	<0.001

Note: HR, hazard ratio; CI, confidence interval; PFS, progression-free survival. The three variable-selection strategies converged on the identical three-variable model, confirming TOP2A mRNA as a robust independent prognostic factor for PFS (n = 154 patients, 19 progression events).
