# Supplementary material for: TOP2A Overexpression as a Prognostic Biomarker in High-Grade Meningioma: An Integrated Single-Cell and Bulk Transcriptomic Study
Source: Int J Mol Sci. 2026 Oct 10;27(20):8984. doi: 10.3390/ijms27208984 (PMC13704434; doi:10.3390/ijms27208984)
Supplement: Supplementary file 1 [file ijms-27-08984-s001.zip › Supplementary Table S9_Forced_Covariate_Sensitivity.pdf]

Supplementary Table S9. Sensitivity analysis: multivariable Cox model with all covariates forced in irrespective of univariate P value

Progression-free survival, n = 154 patients; 19 progression events (12.3%).

Variable	HR (95% CI)	P value
TOP2A mRNA (per $\log_2$ unit)	1.65 (1.19–2.30)	2.6×10^{-3}
WHO grade (per grade)	2.54 (0.89–7.23)	0.081
Simpson grade (per grade)	2.42 (1.49–3.92)	3.3×10^{-4}
Ki-67 (per 10%)	1.32 (0.65–2.67)	0.437
Age (per year)	0.99 (0.95–1.03)	0.507
Adjuvant radiotherapy (yes vs no)	0.36 (0.09–1.39)	0.138

Note: TOP2A mRNA, WHO grade, Simpson grade, age, adjuvant radiotherapy and Ki-67 were all entered simultaneously, irrespective of their univariate P value. Overall model: likelihood-ratio test χ^2 = 37.44 (df = 6), P = 1.45×10^{-6}; concordance index (C-index) = 0.860; AIC = 145.01. HR, hazard ratio; CI, confidence interval. P values are from the Wald test.

Model stability: With 19 progression events across six covariates (3.2 events per variable), this model sits at the lower limit of what the data can support, and the resulting estimates are correspondingly imprecise. Three checks were therefore performed. (i) Leave-one-covariate-out refitting: removing each of the other five covariates in turn left the TOP2A estimate essentially unchanged (HR range 1.52–1.69; P from 1.1×10^{-3} to 8.8×10^{-3}). (ii) Bootstrap resampling (1,000 replicates, 942 converged): median TOP2A HR = 1.67, 95% percentile interval 0.82–2.78, the width of this interval reflecting the low event count. (iii) Ridge (L2) penalised fitting with penaliser 0.1: TOP2A HR = 1.30 (95% CI 1.03–1.63), P = 0.026. The direction and significance of the TOP2A association were therefore preserved under all three checks, although the confidence intervals in this fully adjusted model are wide.
